# Prevalence of Mental Health Problems and Factors Associated with Psychological Distress in Mountain Exercisers: A Cross-Sectional Study in Austria

**DOI:** 10.3389/fpsyg.2017.01237

**Published:** 2017-07-20

**Authors:** Martin Niedermeier, Arnulf Hartl, Martin Kopp

**Affiliations:** ^1^Department of Sport Science, University of Innsbruck Innsbruck, Austria; ^2^Institute of Ecomedicine, Paracelsus Medical University Salzburg, Austria

**Keywords:** depression, anxiety, physical activity, exercise, affective responses

## Abstract

Knowledge about potential protective factors against mental health problems is highly needed. Regular physical activity (PA) in an outdoor environment, like mountain exercising, might reduce psychological distress. Therefore, the aims of the present study were to assess the prevalence of mental health problems in mountain exercisers and to detect factors associated with psychological distress. In a cross-sectional design, we collected self-reported data of 1,536 Austrian mountain exercisers. The prevalence of mental health problems and psychological distress (Kessler Psychological Distress Scale), the level of PA International Physical Activity Questionnaire, and affective valence during PA (Feeling Scale) were obtained. Stepwise multiple linear regression analysis was conducted to assess factors influencing psychological distress. The prevalence of mental health problems in Austrian mountain exercisers was 14%. Health-enhancing PA level and higher affective valence during PA were significantly associated with lower psychological distress. Minimal PA level was not significantly associated with lower psychological distress compared to inactive PA level. Marital status, education, alpine association membership, and body mass index did not show a significant influence on psychological distress. The prevalence of mental health problems seems to be lower in Austrian mountain exercisers compared to the European population. A health-enhancing PA level and affective valence increasing forms of PA were shown to be associated with lower psychological distress. Results might lead to interventional studies focusing on the potential of outdoor PA, e.g., mountain exercise, as an adjunct treatment in people at risk or with mental health problems.

## Introduction

There is a high prevalence of mental health problems in European countries: with 38% of the European citizens, 164.8 million persons suffer from a mental health problem each year (Wittchen et al., 2011). Mental health problems are also associated with higher prevalence in risk factors for chronic diseases (Prince et al., 2007). This leads to a large burden for affected persons, but also to enormous expenses of the public health care system. The direct healthcare costs for anxiety disorders in Europe accounted for more than eight billion € in the year 2010 (Gustavsson et al., 2011). However, this number does not include the costs of other frequent mental health problems (e.g., mood disorders, psychotic disorders, American Psychiatric Association, 2000) or indirect costs of mental health problems (e.g., disability benefit claims). The percentage of disability benefit claims related to mental health problems increased from 10% of all disability benefit claims in 1995 to 35% in 2013 (OECD, 2015). In Austria, the combined direct and indirect costs for mental health problems account for 3.6% of the gross domestic product (OECD, 2015).

Given this large economic burden of mental health problems, cost-effective interventions for prevention and treatment are urgently needed. There is empirical evidence that regular physical activity (PA) can serve as an effective preventive behavior as well as an intervention for mental health problems (Cooney et al., 2013; Mammen and Faulkner, 2013). Despite this, more than 65% of the European population do not meet the criteria for health-enhancing PA (Sjöström et al., 2006). Health-enhancing PA is defined by approximately 1.5 to 2 h of being active throughout the day based on standard scoring criteria of the International Physical Activity Questionnaire^1^. Alongside the physiological benefits, there is a vast knowledge about mood improvement through PA. Affective valence, defined as the degree of pleasure (or displeasure) in a specific situation (Ekkekakis et al., 2008), may have a bidirectional effect. On one hand, affective valence can be influenced positively by PA with moderate intensity (Ekkekakis et al., 2008; Deslandes, 2014). On the other hand, affective valence plays a key role in maintaining PA (Williams et al., 2008; Rhodes and Kates, 2015), i.e., forms of PA leading to a higher affective valence are more likely to be maintained. Being physically active in an outdoor environment seems to have synergetic effects on affective valence compared to being active indoors (Pretty et al., 2005; Barton and Pretty, 2010; Thompson Coon et al., 2011; Frühauf et al., 2016; Niedermeier et al., 2017). Consequently, especially outdoor PA might affect the maintenance of PA positively and might have the potential to reduce the prevalence of mental health problems (Mammen and Faulkner, 2013).

In alpine areas, mountain exercising, a form of outdoor PA, which plays a key role in PA behavior (Bourdeau et al., 2002). In 2005, nearly 45% of the Austrian population above the age of 15 years was practicing (mountain) hiking or climbing/mountaineering, which are the most prominent representative mountain sport disciplines (Kuratorium für Verkehrssicherheit, 2005; Statistik Austria, 2016). Additionally, mountain exercise is performed by millions of tourists, who are regularly visiting mountainous regions. In 1999, more than 40 million of mountain tourists were estimated in the entire Alps (Burtscher, 1999). Repeated bouts of mountain hiking with moderate intensity showed positive effects on mental health related psychological parameters, both in healthy participants and in patients with metabolic syndrome (Schobersberger et al., 2010; Niedermeier et al., 2017). Furthermore, mountain exercising might also be used in patient populations with mental health problems. Recently, mountain hiking has been effectively used as a treatment option for suicide patients and was recommended as an adjunct to conventional therapy (Sturm et al., 2012).

Overall, mountain exercise as a frequently conducted form of PA in alpine regions may have relevant potential in the prevention and treatment of mental health problems, but at this stage of research, more knowledge based on epidemiological data about potential protective effects of this special form of PA is warranted. Therefore, the aims of the present study were (a) to assess the prevalence of mental health problems in mountain exercisers and (b) to detect potential factors associated with psychological distress in mountain exercisers. We hypothesized that a high level of PA and high affective valence during PA might be associated with lower psychological distress in mountain exercisers.

## Materials and Methods

### Design

The study was conducted in a cross-sectional design. The study protocol was approved by the Board for Ethical Questions in Science of the University of Innsbruck in accordance with the Declaration of Helsinki (No. 25/2015, date: 17.06.2015).

### Sample and Procedure

We collected the data via web-based questionnaire. Information about psychological distress, PA level, health status, affective valence to PA, quality of life and socio-demographic data was collected in 49 questions. The questionnaire was distributed by the largest Austrian Alpine Association to ensure collecting a representative sample of mountain exercisers and via electronic networks of the Paracelsus Medical University web site to recruit mountain exercisers without membership in an Alpine Association. Incentives related to mountain sports were provided to increase the participation of mountain exercisers.

Inclusion criteria were permanent residence in Austria, age 18 years and above, and being mountain exerciser. To our knowledge, there is no clear definition of mountain exercise or mountain sports in literature. However, the largest Austrian mountain sports association summarizes “the vast diversity of modern mountain sports” (Austrian Alpine Association, 2002) and includes the following disciplines: hiking and trekking, climbing via ferratas, classic mountaineering, winter mountaineering (ski or snowboard), all types of climbing (bouldering, climbing on artificial objects, crag climbing, continuous climbing, bigwall/aid climbing, alpine climbing, adventure climbing, sport climbing, super-alpine climbing, expedition climbing). A participant was considered as a mountain exerciser, when the participant was exercising in at least one of the disciplines. Because of the low prevalence of bigwall/aid climbing, super-alpine climbing, and expedition climbing in the Alpine region, we did not ask for these types of mountain exercise.

Initially, there were data of 2,270 subjects (female: 47%), of which 99% (2,244/2,270) were defined as mountain exercisers. Out of 2,244 mountain exercisers (female: 47%), 15% (336) have been excluded because of non-Austrian permanent residence, 1% (15) due to age less than 18 years, 16% (357) due to missing data in covariates (*n* = 302 in PA, *n* = 50 in monthly income, *n* = 5 in affective valence during PA). The final data set consisted of 1,536 mountain exercisers with complete data in all relevant variables.

### Measurements

#### Psychological Distress and Prevalence of Mental Health Problems

The level of non-specific psychological distress was assessed by the Kessler Psychological Distress Scale (K10, Kessler et al., 2002). On the 10-item scale, participants had to rate how often they felt distressed on 5-point Likert scale from “none of the time” (0) to “all of the time” (5) in the last 30 days. All items were summed up to get a total score ranging from 10 to 50. Higher scores indicate a higher level of psychological distress. The K10 showed excellent internal consistency (Cronbach’s α = 0.93) and good convergent validity (*r* = 0.76 to 0.99, Kessler et al., 2002). Internal consistency in the present sample was high (Cronbach’s α = 0.84).

The level of psychological distress shows a strong relationship to mental health problems (Kessler et al., 2002). Consequently, the K10 was used both as a screening tool for mental health problems and as an assessment for the level of psychological distress. The K10 shows good discrimination abilities between cases with mental health problems and non-cases according to the Diagnostic and Statistical Manual of Mental Disorders 4th edition (American Psychiatric Association, 2000) and was shown to be superior compared to other screening scales (Cornelius et al., 2013). The Receiver Operating Characteristic for the discrimination showed values of 0.87 to 0.88 and 0.86, respectively (Kessler et al., 2002; Hides et al., 2007). In accordance to previous studies, subjects with a K10 total score of 10–19 were defined as likely to be well and subjects with K10 total score of 20 and above as likely to have mental health problems (Australian Bureau of Statistics, 2001; Vasiliadis et al., 2015).

#### Self-rated Physical Activity

The level of PA was assessed with the short form of the International Physical Activity Questionnaire consisting of 11 items (IPAQ^2^). Participants were asked to rate the frequency and duration of vigorous, moderate, and walking activity during the last 7 days as well as the sitting time per day. Even though there was some controversy about the validity of the IPAQ (Hallal and Victora, 2004; Lee et al., 2011), the IPAQ is widely used in national surveys (Guthold et al., 2008; Bauman et al., 2011; Kopp et al., 2015) and Craig et al. (2003) concluded that the IPAQ showed acceptable validity values (*r* = 0.8).

The IPAQ allows calculating both a continuous score (energy expenditure in multiples of the basic metabolic rate) and a categorical score [inactive, minimally active, and health enhancing physically active (HEPA)]. These categories were based on standard scoring criteria^2^.

#### Affective Valence during Physical Activity

The German version of the Feeling Scale (FS) was used to operationalize affective valence (Hardy and Rejeski, 1989). The bipolar, single item scale consists of 11 answer possibilities ranging from “very good” (+5) to “very bad” (-5) with a neutral answer possibility. The FS was exclusively designed for measurements of affective valence during PA. Discriminant validity was reported for perceived exertion including other development information (Hardy and Rejeski, 1989). Convergent validity was assessed previously with the Self Assessment Manikin and ranged from *r* = 0.41 to 0.88 (Van Landuyt et al., 2000).

Momentary assessed affective valence during PA showed a large variability due to both intra-individual differences (Unick et al., 2015; Sudeck et al., 2016) and due to different environments (Pretty et al., 2005; Barton and Pretty, 2010; Thompson Coon et al., 2011; Ekkekakis, 2015; Frühauf et al., 2016). In this study, affective valence was assessed in general (i.e., beliefs about affective valence, Robinson and Clore, 2002). Two separate questions were used to assess affective valence for indoor PA (e.g., Volleyball, Squash, and Badminton) and outdoor PA (e.g., mountain hiking, climbing, Ski touring). Consequently, the mean value of beliefs about affective valence indoors and outdoors was used to operationalize affective valence during PA. If the subjects were not physically active indoor, only the value for outdoor PA was used.

#### Covariates

The following covariates were collected because of their potential association with psychological distress and mental health problems: sex (male, female), age group in years (18–39, 40–59, 60 and above), body mass index (BMI) in kg/m^2^ (<18.5, 18.5–24.9, 25.0–29.9, 30.0 and above), marital status (no partnership, married/de facto partnership), education (non-college level, college level), membership in Alpine association (yes, no), income group in €/month (<1300, 1300–1799, 1800–2800, >2800) and health related quality of life (HRQoL). HRQoL was assessed by the EQ-5D-5L (EuroQol Group, 1990). The EQ-5D-5L is a 5-item-questionnaire and covers five dimensions of quality of life: mobility, self-care, usual activities, pain/discomfort, and anxiety/depression. The response mode consists of a 5-point Likert-scale. An index for all dimensions combined is calculated ranging from lowest quality of life (0.000) to highest quality of life (1.000). The EQ-5D-5L showed acceptable convergent validity values, *r* = 0.43 to 0.61 (Janssen et al., 2013).

### Statistical Analyses

All statistical analyses were performed using SPSS v. 23 (IBM, New York, NY, United States). To assess relevant associations to psychological distress, the total sample was divided in two groups. One group was defined as likely to be well (K10: 10–19) and one group was defined as likely to have mental health problems (K10: 20 and above) (Australian Bureau of Statistics, 2001; Vasiliadis et al., 2015). Prior to multiple linear regression analysis, all variables were tested on differences in the two groups to assess relevant predictors of psychological distress. Due to non-normal distribution of the variables (as assessed by Shapiro–Wilk test), Mann–Whitney *U* test was used to find differences between the two groups for metric variables. For categorical variables, χ^2^-methods were used. Bonferroni correction was applied due to multiple testing on differences. Consequently, *p*-values less than 0.005 were considered as significant for Mann–Whitney *U* test and χ^2^-test.

Stepwise multiple linear regression calculations were used to model psychological distress (dependent variable). Step 1 included all relevant demographic covariates differing between the two groups. In step 2, HRQoL was added. In step 3, the PA level was added. The final step 4 included all previous variables and the affective valence during PA.

As expected in a non-clinical sample (Andrews and Slade, 2001), the values of psychological distress were considerably skewed, and so were the values of HRQoL and affective valence during PA. In the regression model, the residuals were not normally distributed. Consequently, the variables psychological distress, HRQoL and affective valence during PA were log-transformed.

The level of significance was set at *p* < 0.05 (two-tailed). Data were presented as median, interquartile range (Mdn, IQR) and percentage (frequency) unless otherwise stated.

## Results

### Prevalence of Mental Health Problems and Group Differences

The overall prevalence of mental health problems in mountain exercisers was 14% (221). The 9% (144) were categorized as likely to have mild or moderate mental health problems. The 5% (77) were categorized as likely to have severe mental health problems. **Table 1** shows the frequencies of the covariates’ categories in subjects who were likely to be well and subjects who were likely to have mental health problems, respectively. Subjects who were likely to have mental health problems showed a significantly higher amount of women, of younger participants, of participants with low income, and a lower amount of HEPA people.

**Table 1:** Subject characteristics in the total sample and between participants who were likely to be well and who were likely to have mental health problems.

Variable	Total sample (*n* = 1536)	Likely to be well (*n* = 1315)	Likely to have mental health problems (*n* = 221)	*P*^3^
Sex, female	703 (46%)	574 (44%)	129 (58%)	<0.001
Marital status, partnership	446 (29%)	382 (29%)	64 (29%)	0.978
Education, college	776 (51%)	662 (50%)	114 (52%)	0.835
Membership in Alpine association, yes	1342 (87%)	1155 (88%)	187 (85%)	0.183
**Age group, years**				
18–39	693 (45%)	567 (43%)	126 (57%)	
40–59	600 (39%)	519 (39%)	81 (37%)	
60 and above	243 (16%)	229 (17%)	14 (6%)	<0.001
**BMI^1^ group, kg/m^2^**				
<18.5	39 (3%)	33 (3%)	6 (3%)	
18.5–24.9	1118 (73%)	949 (72%)	169 (76%)	
25–29.9	329 (21%)	287 (22%)	42 (19%)	
30 and above	50 (3%)	46 (3%)	4 (2%)	0.412
**Income, €/month**				
<1300	270 (18%)	204 (16%)	66 (30%)	
1300–1799	296 (19%)	240 (18%)	56 (25%)	
1800–2800	474 (31%)	427 (32%)	47 (21%)	
>2800	496 (32%)	444 (34%)	52 (24%)	<0.001
**Activity level**				
Inactive	188 (12%)	149 (11%)	39 (18%)	
Minimally active	438 (29%)	356 (27%)	82 (37%)	
HEPA^2^	910 (59%)	810 (62%)	100 (45%)	<0.001


*^1^BMI, Body mass index; ^2^HEPA, health enhancing physically active; ^3^p, p-value of χ^2^-test*.

The variables HRQoL and affective valence during PA showed small, but significant differences between the two groups. HRQoL was significantly lower in subjects who were likely to have mental health problems (Mdn: 0.909, IQR: 0.089) compared to subjects who were likely to be well (Mdn: 1.000, IQR: 0.090), *p* < 0.001. Affective valence during PA was significantly lower in subjects who were likely to have mental health problems (Mdn: 3.5, IQR: 1.5) compared to subjects who were likely to be well (Mdn: 4.0, IQR: 1.5), *p* < 0.001.

Marital status, education, membership in an alpine association, and BMI group did not significantly differ in the two groups and were therefore excluded from the linear regression.

### Regression Analysis of Psychological Distress

**Table 2** shows the results of the four steps of the linear regression. All models were statistically significant. The final model explained 18% of the variance, *F*(10,1525) = 33.84, *p* < 0.001. Protecting sociodemographic factors against psychological distress were being male, being older, and a higher monthly income. Higher HRQoL, higher level of PA, and higher affective valence during PA were associated with lower psychological distress.

**Table 2:** Stepwise linear regression results for psychological distress.

Variable	*n*	Psychological distress Median (IQR^1^)	β^2^	*T*	*P*^3^	*R*^2^ corr. ^4^	Δ*R*^2^ corr. ^5^
**STEP 1**
Sex
Female	703	15 (5)	1
Male	833	14 (4)	-0.09	–3.70	<0.001
Age group, years
18–39	693	15 (5)	1
40–59	600	14 (4)	-0.15	-5.81	<0.001
60 and above	243	13 (4)	-0.25	-9.38	<0.001
Income, €/month
<1300	270	16 (5)	1
1300–1799	296	15 (5)	-0.03	-1.02	0.306
1800–2800	474	14 (5)	-0.14	-4.07	<0.001
>2800	496	14 (4)	-0.17	-5.05	<0.001	0.09
**STEP 2**
HRQoL^6^			-0.21	-8.69	<0.001	0.15	0.06
**STEP 3**
Activity level
Inactive	188	15 (6)	1
Minimally active	438	15 (5)	-0.01	-0.25	0.806
HEPA^7^	910	14 (5)	-0.10	-2.86	0.004	0.15	0.01
**STEP 4**
Affective valence			-0.15	-6.43	<0.001	0.18	0.03


*^1^IQR, interquartile range; ^2^β, standardized regression coefficient; ^3^p, p-value of t-test; ^4^ R^2^ corr., variance explained by the model (corrected value); ^5^ΔR^2^ corr., change in variance explained by the model (corrected value); ^6^HRQoL, Health Related Quality of Life; ^7^HEPA, Health Enhancing Physically Active*.

## Discussion

### Major Findings

To the best of our knowledge, this was the first study to show the prevalence of mental health problems in the specific population of mountain exercisers. The major findings in this study were, that (1) being male, older age, higher income, higher activity level, higher HRQoL and higher affective valence during PA were associated with lower psychological distress. (2) Psychological distress was not significantly associated with membership in alpine association and weight status. (3) The prevalence of mental health problems in mountain exercisers seems to be lower compared to the European population.

### Associations with Psychological Distress

Sex and income were significant sociodemographic covariates to psychological distress, which is consistent with the existing literature (Lipton et al., 2000; Prince et al., 2007; Wittchen et al., 2011; OECD, 2015). Also in mountain exercisers, being male is associated with lower psychological distress. Our results also confirmed previous findings in national and international surveys (Atlantis and Ball, 2007; Wittchen et al., 2011) that the prevalence of mental health problems and high psychological distress was dependent on the income of the participants.

Weight status was not shown to be a significantly associated to psychological distress in our sample, which is contrary to previous findings (Lipton et al., 2000; Atlantis and Ball, 2007; OECD, 2015). Atlantis and Ball (2007) were able to show higher rates of medium and high psychological distress in underweight and obese people. It might be concluded, that in mountain exercisers psychological distress is independent of weight status. However, this conclusion has to be treated with caution, because there were only few cases of underweight/obesity in the present sample of mountain exercisers (combined *n* = 89). It might be a more convincing conclusion that the percentage of underweight/obese people is low in mountain exercisers. Indeed, the prevalence of underweight/obesity (6%) was low compared to the Austrian population (17%) (Statistik Austria, 2016). When the prevalence of medium/high psychological distress was compared in acceptable (BMI: 18.5–24.9 kg/m^2^) and overweight category (BMI: 25.0–29.9 kg/m^2^), our results are consistent with Atlantis and Ball (2007) who did not report a difference in these two groups.

Interestingly, there was no significant difference in psychological distress between inactive and minimally active mountain exercisers. Only when PA level was increased to HEPA category there was a significant psychological distress reducing effect. This finding strengthens the recommendation (also from a mental health point of view) to increase PA level in physically inactive and minimally active persons to HEPA category.

Another important finding was that higher affective valence during PA was associated with lower psychological distress. When the standardized regression coefficients of affective valence during PA and income were compared it can even be concluded that affective valence during PA is nearly equally important as income. It has been shown previously that affective valence during PA play a major role in both starting a PA program (Gollwitzer, 1996; Hall et al., 2002; Ekkekakis et al., 2004, 2010) and maintaining a physically active behavior (Williams et al., 2008; Rhodes and Kates, 2015). The present study provides further evidence that it is important to focus on affective valence during PA and to promote PA that shows positive effects on affective valence.

We expected membership in an alpine association would be associated with lower psychological distress due to social support provided by regular activities in alpine associations, which could not be confirmed in our study. Other authors were able to show a stress-buffering effect of social support (Bovier et al., 2004) and a reduction in depressive symptoms due to voluntary group membership (Rietschlin, 1998). It might be concluded that the degree of social support is comparable in alpine association members and in the peer groups of non-members. Psychological distress in mountain exercisers might be more depending on the social support during mountain exercise *per se* and less on membership. However, this speculation has to be proved in future studies.

### Comparison of Mountain Exercisers with General Population

The prevalence of mental health problems in mountain exercisers was lower (14%) compared to values of 38% (total population European Union, Wittchen et al., 2011), 20% (working population Europe, OECD, 2015), and 25% (Germany, Bijl et al., 2003; Kessler et al., 2009). When mountain exercisers were compared to the Austrian Health Survey (representative study for the Austrian population) in the significant covariates, mountain exercisers showed a younger age (median age group: 40 to 44 years vs. 45 to 49 years, Kopp et al., 2015). Since younger age was related to higher scores in psychological distress, this should result in an even larger rate of mental health problems in the sample of mountain exercisers. However, this difference might be compensated by a different percentage of female population (46% vs. 54%, Kopp et al., 2015). It has been shown previously that women show higher rates of mental health problems (Wittchen et al., 2011). There were also small differences in the BMI and in the monthly income. Mountain exercisers showed a smaller mean BMI (23 vs. 25 kg/m^2^) and a higher median monthly income (1800–2300€ vs. 1501–1800€). No differences in marital status were observed (29% vs. 29% partnership). The PA levels showed comparable percentages in the three categories inactive (present study: 12% vs. Austrian population 17%), minimally active (29% vs. 27%), and health-enhancing physically active (59% vs. 66%, Kopp et al., 2015).

### Limitations

The following limitations have to be considered when interpreting the findings of the present study: Firstly, we cannot exclude a selection bias in the present study. It is known from other studies that the outcome in mental health determinants differs between responders and non-responders (de Winter et al., 2005). Since, we provided incentives related to mountain sports when the questionnaire was completed, we hoped to increase the participation of mountain exercisers with low confidence. Secondly, the explained variance of the final model was low. It remains doubtful if all relevant variables were included in the model. Consequently, we put more attention on the comparison of the associate variables of psychological distress and less attention on the total model. Thirdly, we did not use a clinical measurement of mental health problems (e.g., diagnostic interview, Kessler and Ustün, 2004). Even though the Kessler Scale for Psychological Distress is a widely used screening tool for mental health problems (Mitchell and Beals, 2011; Vasiliadis et al., 2015), the prevalence rates of mental health problems may be not reliable in the present study. Fourthly, due to the cross-sectional design, no comparison of the results could be done with a control group doing indoor PA or being sedentary. Furthermore, we are not able to answer the question about a possible causal effect of the level of PA and affective valence during PA on psychological distress. It remains unclear whether the level of PA affects psychological distress, whether psychological distress affects the level of PA or whether there are mutual influences of the different variables. Prospective studies in the mountain exerciser population are needed to clarify these effects.

## Conclusion

The present study showed that being physically active in HEPA category and experiencing high affective valence during PA seem to be associated with lower psychological distress in mountain exercisers. Obtained results should lead to prospective longitudinal studies focusing on the prevention of mental health problems by outdoor PA on one hand as well as testing the potential of mountain PA as an adjunct treatment in people with mental health problems on the other hand. Furthermore, the results could be helpful for health professionals for PA recommendations for the prevention of mental health problems by including outdoor exercise as an interesting alternative to traditional indoor exercises.

## Author Contributions

All authors contributed to the study design. AH performed the data collection. MN performed the statistical analysis and drafted the manuscript. All authors critically revised the drafted manuscript and provided final approval of the version of the manuscript for publication. All authors agree to be accountable for all aspects of the work ensuring that questions related to the accuracy or integrity of any part of the work are appropriately investigated and resolved.

## Conflict of Interest Statement

The authors declare that the research was conducted in the absence of any commercial or financial relationships that could be construed as a potential conflict of interest. The reviewer CK and handling Editor declared their shared affiliation, and the handling Editor states that the process nevertheless met the standards of a fair and objective review.

## Footnotes

1 http://www.ipaq.ki.se

2 http://www.ipaq.ki.se/

## References

[B1] American Psychiatric Association (2000). *Diagnostic and Statistical Manual of Mental Disorders: DSM-IV-TR*. Arlington County, VA: American Psychiatric Association.

[B2] Andrews G. Slade T. (2001). Interpreting scores on the Kessler Psychological Distress Scale (K10). *Aust. N. Z. J. Public Health* 25 494–497. 10.1111/j.1467-842X.2001.tb00310.x 11824981

[B3] Atlantis E. Ball K. (2007). Association between weight perception and psychological distress. *Int. J. Obes.* 32 715–721. 10.1038/sj.ijo.0803762 18408736

[B4] Australian Bureau of Statistics (2001). *Information Paper: Use of the Kessler Psychological Distress Scale in ABS Health Surveys [Online]*. Canberra, ACT: Australian Bureau of Statistics.

[B5] Austrian Alpine Association (2002). *Tyrol Declaration to Best Practice in Mountain Sports [Online].* Available at: https://theuiaa.org/declarations/tyrol-declaration/ [accessed December 24 2015].

[B6] Barton J. Pretty J. (2010). What is the best dose of nature and green exercise for improving mental health? a multi-study analysis. *Environ. Sci. Technol.* 44 3947–3955. 10.1021/es903183r 20337470

[B7] Bauman A. Ainsworth B. E. Sallis J. F. Hagströmer M. Craig C. L. Bull F. C. (2011). The descriptive epidemiology of sitting. A 20-country comparison using the International Physical Activity Questionnaire (IPAQ). *Am. J. Prev. Med.* 41 228–235. 10.1016/j.amepre.2011.05.003 21767731

[B8] Bijl R. V. de Graaf R. Hiripi E. Kessler R. C. Kohn R. Offord D. R. (2003). The prevalence of treated and untreated mental disorders in five countries. *Health Aff.* 22 122–133. 10.1377/hlthaff.22.3.122 12757277

[B9] Bourdeau P. Corneloup J. Mao P. (2002). Adventure sports and tourism in the French mountains: dynamics of change and challenges for sustainable development. *Curr. Issues Tour.* 5 22–32. 10.1080/13683500208667905

[B10] Bovier P. A. Chamot E. Perneger T. V. (2004). Perceived stress, internal resources, and social support as determinants of mental health among young adults. *Q. Life Res.* 13 161–170. 10.1023/B:QURE.0000015288.43768.e4 15058797

[B11] Burtscher M. (1999). Höhenkopfschmerz: epidemiologie, pathophysiologie, therapie und prophylaxe. [High altitude headache: epidemiology, pathophysiology, therapy and prophylaxis]. *Wien. Klin. Wochenschr.* 111 830–836. 10586487

[B12] Cooney G. M. Dwan K. Greig C. A. Lawlor D. A. Rimer J. Waugh F. R. (2013). Exercise for depression. *Cochrane Database Syst. Rev.* 9:CD004366 10.1002/14651858.CD004366.pub6 PMC9721454 24026850

[B13] Cornelius B. L. Groothoff J. W. van der Klink J. J. Brouwer S. (2013). The performance of the K10 K6 and GHQ-12 to screen for present state DSM-IV disorders among disability claimants. *BMC Public Health* 13:128 10.1186/1471-2458-13-128 PMC3575398 23402478

[B14] Craig C. L. Marshall A. L. Sjöström M. Bauman A. E. Booth M. L. Ainsworth B. E. (2003). International physical activity questionnaire: 12-country reliability and validity. *Med. Sci. Sports Exerc.* 35 1381–1395. 10.1249/01.mss.0000078924.61453.fb 12900694

[B15] de Winter A. F. Oldehinkel A. J. Veenstra R. Brunnekreef J. A. Verhulst F. C. Ormel J. (2005). Evaluation of non-response bias in mental health determinants and outcomes in a large sample of pre-adolescents. *Eur. J. Epidemiol.* 20 173–181. 10.1007/s10654-004-4948-6 15792285

[B16] Deslandes A. C. (2014). Exercise and mental health: what did we learn in the last 20 years? *Front. Psychiatry* 5:66 10.3389/fpsyt.2014.00066 PMC4055854 24982639

[B17] Ekkekakis P. (2015). Honey, I shrunk the pooled SMD! Guide to critical appraisal of systematic reviews and meta-analyses using the Cochrane review on exercise for depression as example. *Mental Health Phys. Act.* 8 21–36. 10.1016/j.mhpa.2014.12.001

[B18] Ekkekakis P. Backhouse S. H. Gray C. Lind E. (2008). Walking is popular among adults but is it pleasant? A framework for clarifying the link between walking and affect as illustrated in two studies. *Psychol. Sport Exerc.* 9 246–264. 10.1016/j.psychsport.2007.04.004

[B19] Ekkekakis P. Hall E. E. Petruzzello S. J. (2004). Practical markers of the transition from aerobic to anaerobic metabolism during exercise: rationale and a case for affect-based exercise prescription. *Prevent. Med.* 38 149–159. 10.1016/j.ypmed.2003.09.038 14715206

[B20] Ekkekakis P. Lind E. Vazou S. (2010). Affective responses to increasing levels of exercise intensity in normal-weight, overweight, and obese middle-aged women. *Obesity* 18 79–85. 10.1038/oby.2009.204 19556979

[B21] EuroQol Group (1990). EuroQol-a new facility for the measurement of health-related quality of life. *Health Policy* 16 199–208. 10109801 10.1016/0168-8510(90)90421-9

[B22] Frühauf A. Niedermeier M. Elliott L. R. Ledochowski L. Marksteiner J. Kopp M. (2016). Acute effects of outdoor physical activity on affect and psychological well-being in depressed patients – A preliminary study. *Ment. Health Phys. Act.* 10 4–9. 10.1016/j.mhpa.2016.02.002

[B23] Gollwitzer P. M. (1996). “The volitional benefits of planning,” in *The Psychology of Action. Linking Cognition and Motivation to Bahavior* eds Gollwitzer P. M. Bargh J. A. (New York, NY: Guilford Press) 287–312.

[B24] Gustavsson A. Svensson M. Jacobi F. Allgulander C. Alonso J. Beghi E. (2011). Cost of disorders of the brain in Europe 2010. *Eur. Neuropsychopharmacol.* 21 718–779. 10.1016/j.euroneuro.2011.08.008 21924589

[B25] Guthold R. Ono T. Strong K. L. Chatterji S. Morabia A. (2008). Worldwide variability in physical inactivity: A 51-country survey. *Am. J. Prev. Med.* 34 486–494. 10.1016/j.amepre.2008.02.013 18471584

[B26] Hall E. E. Ekkekakis P. Petruzzello S. J. (2002). The affective beneficence of vigorous exercise revisited. *Br. J. Health Psychol.* 7 47–66. 10.1348/135910702169358 14596717

[B27] Hallal P. C. Victora C. G. (2004). Reliability and validity of the International Physical Activity Questionnaire (IPAQ). *Med. Sci. Sports Exerc.* 36 556 10.1249/01.mss.0000117161.66394.07 15076800

[B28] Hardy C. J. Rejeski W. J. (1989). Not what, but how one feels: the measurement of affect during exercise. *J. Sport Exerc. Psychol.* 11 304–317. 10.1123/jsep.11.3.304

[B29] Hides L. Lubman D. I. Devlin H. Cotton S. Aitken C. Gibbie T. (2007). Reliability and validity of the Kessler 10 and patient health questionnaire among injecting drug users. *Aust. N. Z. J. Psychiatry* 41 166–168. 10.1080/00048670601109949 17464695

[B30] Janssen M. F. Pickard A. S. Golicki D. Gudex C. Niewada M. Scalone L. (2013). Measurement properties of the EQ-5D-5L compared to the EQ-5D-3L across eight patient groups: a multi-country study. *Qual. Life Res.* 22 1717–1727. 10.1007/s11136-012-0322-4 23184421 PMC3764313

[B31] Kessler R. Andrews G. Colpe L. J. Hiripi E. Mroczek D. K. Normand S.-L. (2002). Short screening scales to monitor population prevalences and trends in non-specific psychological distress. *Psychol. Med.* 32 959–976. 10.1017/S0033291702006074 12214795

[B32] Kessler R. C. Aguilar-Gaxiola S. Alonso J. Chatterji S. Lee S. Ormel J. (2009). The global burden of mental disorders: an update from the WHO World Mental Health (WMH) surveys. *Epidemiol. Psichiatr. Soc.* 18 23–33. 10.1017/S1121189X00001421 19378696 PMC3039289

[B33] Kessler R. C. Ustün T. B. (2004). The world mental health (WMH) survey initiative version of the world health organization (WHO) composite international diagnostic interview (CIDI). *Int. J. Methods Psychiatr. Res.* 13 93–121. 10.1002/mpr.168 15297906 PMC6878592

[B34] Kopp M. Burtscher M. Kopp-Wilfling P. Ruedl G. Kumnig M. Ledochowski L. (2015). Is there a link between physical activity and alcohol use? *Subst. Use Misuse* 50 546–551. 10.3109/10826084.2014.980957 25458711

[B35] Kuratorium für Verkehrssicherheit (2005). *Freizeitunfallstatistik 2005 [Online].* Available at: http://unfallstatistik.kfv.at/uploads/media/Freizeitunfallstatistik_2005.pdf [accessed December 24 2015].

[B36] Lee P. H. Macfarlane D. J. Lam T. H. Stewart S. M. (2011). Validity of the international physical activity questionnaire short form (IPAQ-SF): a systematic review. *Int. J. Behav. Nutr. Phys. Act.* 8:115 10.1186/1479-5868-8-115 PMC3214824 22018588

[B37] Lipton R. Hamelsky S. Kolodner K. Steiner T. Stewart W. (2000). Migraine, quality of life, and depression a population-based case–control study. *Neurology* 55 629–635. 10.1212/WNL.55.5.629 10980724

[B38] Mammen G. Faulkner G. (2013). Physical activity and the prevention of depression: a systematic review of prospective studies. *Am. J. Prev. Med.* 45 649–657. 10.1016/j.amepre.2013.08.001 24139780

[B39] Mitchell C. M. Beals J. (2011). The utility of the kessler screening scale for psychological distress (K6) in two American Indian communities. *Psychol. Assess.* 23 752–761. 10.1037/a0023288 21534694 PMC3150622

[B40] Niedermeier M. Einwanger J. Hartl A. Kopp M. (2017). Affective responses in mountain hiking-A randomized crossover trial focusing on differences between indoor and outdoor activity. *PLoS ONE* 12:e0177719 10.1371/journal.pone.0177719 PMC5433751 28520774

[B41] OECD (2015). *Mental Health and Work.* Paris: OECD Publishing.

[B42] Pretty J. Peacock J. Sellens M. Griffin M. (2005). The mental and physical health outcomes of green exercise. *Int. J. Environ. Health Res.* 15 319–337. 10.1080/09603120500155963 16416750

[B43] Prince M. Patel V. Saxena S. Maj M. Maselko J. Phillips M. R. (2007). No health without mental health. *Lancet* 370 859–877. 10.1016/s0140-6736(07)61238-0 17804063

[B44] Rhodes R. E. Kates A. (2015). Can the affective response to exercise predict future motives and physical activity behavior? A systematic review of published evidence. *Ann. Behav. Med.* 49 715–731. 10.1007/s12160-015-9704-5 25921307

[B45] Rietschlin J. (1998). Voluntary association membership and psychological distress. *J. Health Soc. Behav.* 39 348–355. 10.2307/2676343 9919856

[B46] Robinson M. D. Clore G. L. (2002). Belief and feeling: evidence for an accessibility model of emotional self-report. *Psychol. Bull.* 128 934–960. 10.1037/0033-2909.128.6.934 12405138

[B47] Schobersberger W. Leichtfried V. Mueck-Weymann M. Humpeler E. (2010). Austrian moderate altitude studies (AMAS): benefits of exposure to moderate altitudes (1500-2500 m). *Sleep Breath.* 14 201–207. 10.1007/s11325-009-0286-y 19669819

[B48] Sjöström M. Oja P. Hagströmer M. Smith B. J. Bauman A. (2006). Health-enhancing physical activity across European Union countries: the Eurobarometer study. *J. Public Health* 14 291–300. 10.1007/s10389-006-0031-y

[B49] Statistik Austria (2016). *Population Statistics [Online].* Available at: http://www.statistik.at/web_de/statistiken/menschen_und_gesellschaft/bevoelkerung/bevoelkerungsstruktur/bevoelkerung_nach_alter_geschlecht/index.html [accessed 03 March, 2016].

[B50] Sturm J. Plöderl M. Fartacek C. Kralovec K. Neunhäuserer D. Niederseer D. (2012). Physical exercise through mountain hiking in high-risk suicide patients. A randomized crossover trial. *Acta Psychiatr. Scand.* 126 467–475. 10.1111/j.1600-0447.2012.01860.x 22486584

[B51] Sudeck G. Schmid J. Conzelmann A. (2016). Exercise experiences and changes in affective attitude: direct and indirect effects of in situ measurements of experiences. *Front. Psychol.* 7:900 10.3389/fpsyg.2016.00900 PMC4909746 27378992

[B52] Thompson Coon J. Boddy K. Stein K. Whear R. Barton J. Depledge M. H. (2011). Does participating in physical activity in outdoor natural environments have a greater effect on physical and mental wellbeing than physical activity indoors? a systematic review. *Environ. Sci. Technol.* 45 1761–1772. 10.1021/es102947t 21291246

[B53] Unick J. L. Strohacker K. Papandonatos G. Williams D. O’Leary K. C. Dorfman L. (2015). Examination of the consistency in affective response to acute exercise in overweight and obese women. *J. Sport Exerc. Psychol.* 37 534–546. 10.1123/jsep.2015-0104 26524099 PMC4724861

[B54] Van Landuyt L. M. Ekkekakis P. Hall E. E. Petruzzello S. J. (2000). Throwing the mountains into the lakes: on the perils of nomothetic conceptions of the exercise-affect relationship. *J. Sport Exerc. Psychol.* 22 208–234. 10.1123/jsep.22.3.208

[B55] Vasiliadis H.-M. Chudzinski V. Gontijo-Guerra S. Préville M. (2015). Screening instruments for a population of older adults: the 10-item Kessler psychological distress scale (K10) and the 7-item generalized anxiety disorder scale (GAD-7). *Psychiatry Res.* 228 89–94. 10.1016/j.psychres.2015.04.019 25956759

[B56] Williams D. M. Dunsiger S. Ciccolo J. T. Lewis B. A. Albrecht A. E. Marcus B. H. (2008). Acute affective response to a moderate-intensity exercise stimulus predicts physical activity participation 6 and 12 months later. *Psychol. Sport Exerc.* 9 231–245. 10.1016/j.psychsport.2007.04.002 18496608 PMC2390920

[B57] Wittchen H. U. Jacobi F. Rehm J. Gustavsson A. Svensson M. Jonsson B. (2011). The size and burden of mental disorders and other disorders of the brain in Europe 2010. *Eur. Neuropsychopharmacol.* 21 655–679. 10.1016/j.euroneuro.2011.07.018 21896369

